# Prevalence of burnout and associated factors among general practitioners in Hubei, China: a cross-sectional study

**DOI:** 10.1186/s12889-019-7755-4

**Published:** 2019-12-02

**Authors:** Yong Gan, Heng Jiang, Liqing Li, Yudi Yang, Chao Wang, Jianxin Liu, Tingting Yang, Sampson Opoku, Sai Hu, Hongbin Xu, Chulani Herath, Yuanyuan Chang, Pengqian Fang, Zuxun Lu

**Affiliations:** 1 Department of Social Medicine and Health Management, School of Public Health, Tongji Medical College, Huazhong University of Science and Technology, No. 13 Hangkong Road, Wuhan, 430030 Hubei China; 2 Centre for Alcohol Policy Research, School of Psychology and Public Health, La Trobe University, Melbourne, Victoria Australia; 3 Centre for Health Equity, Melbourne School of Population and Global Health, University of Melbourne, Melbourne, Victoria Australia; 4 Department of Management Science and Engineering, School of Economics and Management, Jiangxi Science and Technology Normal University, Nanchang, Jiangxi China; 5 Department of Nutrition, People’s Hospital of Henan Province, Zhengzhou, Henan China; 6 Department of Medical Records and Statistics, Union Hospital, Tongji Medical College, Huazhong University of Science and Technology, Wuhan, Hubei China; 7 Department of Psychology and Counselling, Faculty of Health Sciences, The Open University of Sri Lanka, Nawala, Nugegoda, Sri Lanka; 8 Academy of Health Policy and Management, School of Medicine and Health Management, Tongji Medical College, Huazhong University of Science and Technology, No. 13 Hangkong Road, Wuhan, Wuhan, 430030 Hubei China; 9 Nanjing Medical University, Nanjing, Jiangsu China

**Keywords:** Burnout, General practitioners, Primary healthcare, Influencing factors

## Abstract

**Background:**

High occupational burnout among general practitioners (GPs) is an important challenge to China’s efforts to strengthen its primary healthcare delivery; however, data to help understand this issue are unavailable. This study aimed to investigate the prevalence of burnout and associated factors among GPs.

**Methods:**

A cross-sectional design was used to collect data from December 12, 2014, to March 10, 2015, with a self-administered structured questionnaire from 1015 GPs (response rate, 85.6%) in Hubei Province, Central China. Burnout was measured using a 22-item Maslach Burnout Inventory-Human Services Survey (MBI-HSS). MBI-HSS scores and frequency were analyzed by the three dimensions of emotional exhaustion (EE), depersonalization (DP), and personal accomplishment (PA). Factors associated with burnout among GPs were estimated using a multiple linear regression model.

**Results:**

Of the respondents, 2.46% had a high level of burnout in all three dimensions, 24.83% reported high levels of EE, 6.21% scored high on DP, and 33.99% were at high risk of PA. GPs who were unmarried, had lower levels of job satisfaction, and had been exposed to workplace violence experienced higher levels of burnout. Intriguingly, no statistically significant associations were found between burnout and the duration of GP practice, age, sex, income, practice setting, and professional level.

**Conclusion:**

This is the first study of occupational burnout in Chinese general practice. Burnout is prevalent among GPs in Hubei, China. Interventions aimed at increasing job satisfaction, improving doctor-patient relationships and providing safer workplace environments may be promising strategies to reduce burnout among GPs in Hubei, China.

## Background

Burnout is a psychological syndrome that is a reaction to the accumulation and long-term negative effect of chronic work-related stress [1]. It is a syndrome characterized by feelings of overextension and the depletion of resources (emotional exhaustion), negative or callous responses to job responsibilities (depersonalization), and feelings of incompetence and a lack of achievement (decreased personal accomplishment) [2]. Burnout has been shown to exert an adverse effect on organizations (e.g., turnover, higher absenteeism, lack of job commitment, and job dissatisfaction), the mental and physical health of healthcare practitioners, and the quality of healthcare delivery [2–7]. Therefore, the primary prevention of occupational burnout should be considered as a public health priority worldwide.

Physicians are particularly vulnerable to experiencing burnout due to heavy workloads and high levels of work-related stresses [8]. A previous study found that physicians were significantly more likely than any other occupational group to experience symptoms of burnout [9]. Additionally, the degrees of burnout vary among medical specialties. General practitioners (GPs) have been found to have higher levels of burnout than physicians in other specialties [9–11]. Previous studies have mainly focused on burnout and its related factors among GPs in Western countries [10, 12–21]. GPs are the gatekeepers of the primary healthcare system. The efficiency, effectiveness and quality of a nation’s healthcare system is directly associated with the quality and quantity of its workforce, including GPs. In China, GPs work in Primary Health Institutions (PHIs), Township Health Centers (THCs), and Community Health Centers (CHCs), and mainly provide general medical services such as diagnosis, treatment and prevention of acute and chronic diseases. In addition, they provide referrals to secondary or tertiary hospitals. According to the 2014 Chinese Medical Doctors Association report, 66% of GPs worked more than 40 h per week [22]. These long working hours present a risk for GPs to develop burnout [4, 23–25].

Recently, shortages of primary healthcare workers and high GP turnover in China have been major issues. The establishment of a GP team is a key strategy to strengthen PHIs. A high level of burnout among Chinese GPs could lead to a reduction in their numbers of GPs and damage to their well-being, which could consequently affect the quality of health care. The above issues could result in serious repercussions on the country’s healthcare system. Few existing studies in China have focused on data on burnout among physicians, and to date, research has not distinguished between GPs or non-GPs [26]. This study aimed to assess the prevalence of burnout and its relevant predictors among GPs who work in PHIs in Hubei, China.

## Methods

### Study participants and settings

A cross-sectional study was conducted in Central China from December 2014 to March 2015. With a total of 12 independent variables included in the multivariable analysis model, to detect a 2% (effect size) difference between two subgroups (e.g., men vs. women or respondents with low job satisfaction vs. high job satisfaction) with 95% predictive power, the ideal sample size was 652 (computed by G-Power 3.1).

A stratified random sample design was used. According to the China Health Statistics Yearbook 2013 [27], there were 2325 PHIs in 17 prefecture-level cities of Hubei Province, and all of them were included in this study. According to the number of GPs and the scales of PHIs, we randomly selected 30% of the on-post GPs with at least 1 year of work experience from each sampled PHI. Among the 3752 eligible GPs, 1186 were randomly invited to participate in a training seminar on quality health care organized by the general practice training center of Hubei Province. After the training session, they were asked to complete a self-administered questionnaire. Overall, 1101 GPs participated in the survey. However, 86 questionnaires were excluded because of missing information on burnout (more than two items). Finally, 1015 eligible GPs were included in the analysis (response rate, 85.6%).

All respondents provided written informed consent, and the study was approved by the Ethics Committee of the Tongji Medical College Institutional Review Board, Huazhong University of Science and Technology (Wuhan, China).

### Questionnaire design and content

The questionnaire was designed based on literature reviews, group discussions, and mock interviews. Furthermore, a pilot study was conducted in Wuhan’s CHCs to improve the questionnaire’s quality. The questionnaire comprised five sections: sociodemographic information, job satisfaction, burnout, workplace violence, and turnover intention. Given the purpose of this study, the data from sections 1, 2, 3 and 4 were included.

### Measurements

Burnout was measured with a standardized scale that consisted of 22 items on a six-point Likert scale ranging from 0 (never) to 6 (every day). Burnout was assessed based on three subscales: emotional exhaustion (EE), depersonalization (DP), and personal achievement (PA) were measured with the 22-item Maslach Burnout Inventory (MBI-HSS) [28]. Higher scores on the EE and DP subscales were positively associated with higher levels of burnout, while PA was inversely associated with burnout.

In the analysis of the prevalence of burnout, we analyzed the subscales as categorical variables. The cut-off points for different categories (low, moderate, and high) of each subscale were defined in the study according to the MBI-HSS scoring guidelines [29]. GPs were categorized as having a high level of burnout if they scored high on EE and DP and low on PA. In this study, the Cronbach’s alpha coefficients for the MBI-HSS, EE, DP, and PA were 0.85, 0.91, 0.87 and 0.90, respectively, suggesting that the overall measurement was reliable.

We used the Chinese version of the Workplace Violence Scale developed by Wang et al. [30] consisting of 5 items on a four-point Likert scale ranging from 0 (never) to 3 (more than 3 times/year). Workplace violence included physical violence (physical and sexual assaults) and nonphysical violence (verbal abuse, threats, and sexual harassment). Workplace violence, as an independent variable of burnout, was recoded into yes (encountering one type of workplace violence) and no (not experiencing any type of workplace violence) for the statistical analyses.

### Search strategy

We searched PubMed for similar studies that were conducted in the field between the 1990s and 2018 to compare the prevalence and scores of burnout among GPs in China and other countries.

### Statistical analysis

Student’s *t* test and a one-way analysis of variance (ANOVA) were applied to examine the differences in burnout and its subscales among groups. Dependent variables (EE, DP, PA, and overall burnout) were treated as continuous variables. A multiple linear regression model was performed to estimate the predictors of GPs’ burnout and its three subscales. Predictive variables such as age (continuous), gender, marital status, education level, work tenure, average monthly income, contract status, professional level, managerial responsibility, practice setting, job satisfaction, and workplace violence were included in the multiple linear regression analysis. All statistical analyses were performed with Statistical Analysis System (SAS) version 9.2 (SAS Institute Inc., Cary, NC, USA). Statistical significance was accepted at the 5% level (*P* < 0.05).

## Results

Table 1 shows the basic characteristics of the study respondents. Of the 1015 respondents, most were married (91.92%) and more than half were men. Half of respondents were 40 years or older, and 52.10% had a bachelor’s degree or above. Among the respondents, the mean time (standard deviation, SD) in the profession was 16.32 (7.90) years. More than half (55.47%) of the respondents worked in general practice settings in urban areas. In total, 62.2% of the respondents reported exposure to workplace violence in the preceding year.

**Table 1:** Burnout score according to socio-demographic and work-related characteristics

	Burnout			EE		DP		PA	
Variables	N (%)	Mean (SD)	*P value*	Mean (SD)	*P value*	Mean (SD)	*P value*	Mean (SD)	*P value*
Age group (years)									
< 30	84 (8.28)	39.00 (20.37)	0.1751	19.71(11.41)	0.0124	4.36 (4.76)	0.0280	33.07 (11.08)	0.0683
30~	405 (39.94)	37.12 (20.28)		19.33 (12.24)		4.21 (4.97)		34.42 (11.20)	
40~	474 (46.75)	35.18 (19.90)		18.54 (11.83)		3.70 (4.65)		35.06 (11.78)	
50~	51 (5.03)	33.04 (19.82)		13.61(12.97)		2.31 (3.34)		30.88 (16.26)	
Sex									
Men	659 (64.99)	37.5 (20.40)	0.0032	19.17 (12.40)	0.0732	4.19 (5.08)	0.0061	33.87 (12.03)	0.0365
Women	355 (35.01)	33.6 (19.3)		17.75 (11.28)		3.34 (4.02)		35.49 (11.28)	
Education level									
Associated degree or vocational diploma^a^	479 (49.90)	35.92 (20.21)	0.6347	17.98 (12.37)	0.0492	3.84 (4.86)	0.6805	33.90 (12.25)	0.1698
Bachelor degree or above	521 (52.10)	36.52 (20.03)		19.48 (11.78)		3.96 (4.69)		34.92 (11.32)	
Marital status									
Unmarried/widow/divorced	67 (6.70)	42.48 (21.19)	0.0076	22.00 (12.15)	0.0196	5.52 (5.49)	0.0128	33.04 (9.88)	0.319
Married	933 (93.30)	35.67 (20.04)		18.44 (12.03)		3.77 (4.67)		34.53 (11.91)	
Contract status									
Permanent	734 (73.18)	35.91 (20.27)	0.3553	18.58 (12.16)	0.5732	3.75 (4.74)	0.0731	34.42 (12.20)	0.7728
Temporary^b^	269 (26.82)	37.24 (19.78)		19.07 (11.87)		4.36 (4.81)		34.19 (10.68)	
Average monthly income (RMB Yuan)									
< 2500	340 (33.76)	37.9 (20.5)	0.1322	19.85 (12.60)	0.0951	4.17 (4.93)	0.3162	34.15 (11.36)	0.7700
2500~	474 (47.07)	35.0 (20.0)		18.04 (11.96)		3.67 (4.68)		34.70 (12.21)	
3500~	193 (19.17)	36.1 (19.6)		18.32 (11.32)		3.97 (4.65)		34.20 (11.58)	
Professional level									
Elementary or less^c^	482 (47.96)	35.84 (20.14)	0.5844	17.90 (11.82)	0.0397	4.18 (5.08)	0.0908	34.24 (11.41)	0.6236
Intermediate or higher^d^	523 (52.04)	36.53 (20.10)		19.47 (12.30)		3.67 (4.45)		34.60 (12.15)	
Has management responsibility									
Yes	332 (34.05)	37.03 (20.89)	0.3196	19.32 (12.61)	0.2649	4.14 (5.23)	0.1677	34.42 (12.20)	0.9954
No	643 (65.95)	35.70 (19.49)		18.41 (11.70)		3.70 (4.33)		34.43 (11.54)	
Practice setting									
CHC	563 (55.47)	35.19 (19.47)	0.0858	18.13 (11.36)	0.0912	3.70 (4.47)	0.1177	34.63 (11.40)	0.5683
THC	452 (44.53)	37.37 (20.82)		19.42 (12.85)		4.15 (5.07)		34.21 (12.25)	
Work tenure (years)	16.32 (7.90)								
< 10	236 (23.32)	38.75 (19.77)	0.0423	19.72 (11.17)	0.3132	4.49 (5.08)	0.0506	33.46 (10.88)	0.1912
10~	385 (38.04)	34.57 (19.88)		18.24 (12.14)		3.53 (4.23)		35.19 (11.41)	
20~	391 (38.64)	36.16 (20.44)		18.54 (12.50)		3.86 (4.99)		34.25 (12.65)	
Workplace violence									
Yes	631 (62.17)	39.98 (20.52)	< 0.0001	21.43 (12.54)	< 0.0001	4.74 (5.29)	< 0.0001	34.19 (10.90)	0.3963
No	384 (37.83)	29.88 (17.70)		14.23 (9.69)		2.51 (3.25)		34.86 (13.10)	

Abbreviations: *CHC* Community health center, *DP* Depersonalization, *EE* Emotion exhaustion, *PA* Personal accomplishment, *SD* Standard deviation, *THC* Township health center

^a^GPs who have acquired associate degree or vocational diploma. An associate degree required 3 years of education in college after graduation from senior middle school (grade year 10 to year 12), or 5 years of education in college after graduation from junior middle school (grade year 7 to year 9). A vocational diploma requires 2 years of education in vocational schools after graduation from senior middle school, or 3 years of education in vocational schools after graduation from junior middle school

^b^Wages and welfares of GPs would be paid by the Chinese government public health services expenditure. GPs were unable to be freely fired off by health institutions

^c^GPs were with junior or without any technical title

^d^GPs were with middle or senior professional title

The participants’ mean scores were 18.70 (SD = 12.06; range = 0–54) on the EE subscale, 3.89 (SD = 4.75; range = 0–30) on the DP subscale, and 34.44 (SD = 11.78; range = 0–48) on the PA subscale. This represents a pattern of moderate EE, low DP, and moderate PA. Higher mean scores of burnout were found among respondents who were male, married, and had experienced workplace violence.

Table 2 shows the prevalence rate of burnout and its subscale among GPs. Among the GPs, 24.83% reported high levels of burnout on EE and 6.21% reported high levels on DP, and 33.99% exhibited reduced feelings of PA. In total, 35% of the respondents scored high for burnout in any dimension, 21% scored high for burnout in at least two dimensions, and 2.46% scored high for all three dimensions.

**Table 2:** Frequency of GPs by degree of burnout (low, moderate and high) with 95% CI in each of the three dimensions

	Burnout in all three dimensions			EE^a^		DP^b^		PA^c^
Variables	*N*	% (95%CI)	*N*	% (95%CI)	*N*	% (95%CI)	*N*	% (95%CI)
Low^d^	277	27.29 (24.57–30.14)	527	51.92 (48.80–55.03)	830	81.77 (79.26–84.10)	467	46.01 (42.91–49.13)
Moderate	713	70.25 (67.33–73.05)	236	23.25 (20.68–25.97)	122	12.02 (10.08–14.18)	203	20.00 (17.58–22.60)
High^e^	25	2.46 (1.60–3.61)	252	24.83 (22.20–27.61)	63	6.21 (4.80–7.87)	345	33.99 (31.08–37.00)

Note: Abbreviations: *DP* Depersonalization, *EE* Emotion exhaustion, *PA* Personal accomplishment

^a^Score ≤ 16 indicated low level; 17 to 26 indicated moderate level; ≥27 indicated high level

^b^Score ≤ 6 indicated low level; 7 to 12 indicated moderate level; ≥13 indicated high level

^c^Score ≥ 39 indicated low level; 32 to 38 indicated moderate level; ≤31 indicated high level

^d^Low degree of burnout was defined by a low score on the subscales for EE and DP, and a high score on the PA subscale

^e^High degree of burnout was defined by a high score on the subscales for EE and DP, and a low score on the PA subscale

The factors associated with GPs’ burnout and the three subscales are presented in Table 3. GPs who were single/divorced-separated, who had a lower level of job satisfaction, and who had experienced workplace violence were more likely to report a higher risk of burnout.

**Table 3:** Multiple linear regression analysis for the association factors with the higher level of burnout and their subscale among GPs (*N* = 922)

Characteristic	Burnout in all three dimensions		EE		DP		PA	
	B (SE)	*P* value	B (SE)	*P* value	B (SE)	*P* value	B (SE)	*P* value
Intercept	88.84 (7.23)	< 0.001	50.65 (4.4)	< 0.001	13.28 (1.79)	< 0.001	23.09 (4.69)	< 0.001
Age (years)	− 0.01(0.22)	0.951	− 0.11(0.13)	0.385	−0.04 (0.05)	0.463	−0.14 (0.14)	0.317
Work tenure (years)	−0.10 (0.19)	0.611	−0.02 (0.11)	0.842	0.01 (0.05)	0.866	0.08 (0.12)	0.509
Job satisfaction	− 1.30 (0.10)	**< 0.001**	−0.65 (4.40)	**< 0.001**	−0.20 (0.02)	**< 0.001**	0.46 (0.06)	**< 0.001**
Sex								
Men	1.95 (1.40)	0.164	−0.13 (0.85)	0.878	0.55 (0.35)	0.111	−1.53 (0.91)	0.093
Women	0.00		0.00		0.00		0.00	
Education level								
Associated degree or vocational diploma^a^	1.43 (1.34)	0.287	−0.07 (0.82)	0.933	−0.02 (0.33)	0.96	−1.52 (0.87)	0.08
Bachelor degree or higher	0.00		0.00		0.00		0.00	
Marital status								
Married	−5.37 (2.46)	**0.03**	−2.86 (1.50)	0.056	−1.17 (0.61)	0.054	1.33 (1.60)	0.404
Unmarried/widow/divorced	0.00		0.00		0.00		0.00	
Contract status								
Permanent	0.25 (1.42)	0.86	0.31 (0.86)	0.723	−0.23 (0.35)	0.51	−0.18 (0.92)	0.846
Temporary^b^	0.00		0.00		0.00		0.00	
Job title								
Elementary or less^c^	−0.13 (1.46)	0.93	−1.96 (0.89)	**0.028**	0.78 (0.36)	**0.03**	−1.04 (0.95)	0.271
Intermediate or higher^d^	0.00		0.00		0.00		0.00	
Practice setting								
THC	−0.35 (1.39)	0.804	0.33 (0.85)	0.702	0.21 (0.34)	0.549	0.88 (0.90)	0.332
CHC	0.00		0.00		0.00		0.00	
Average monthly income								
< 2500	−1.59 (1.83)	0.387	0.06 (1.12)	0.957	−0.68 (0.45)	0.137	0.97 (1.19)	0.414
2500~	−1.03 (1.63)	0.526	−0.12 (0.99)	0.9	−0.42 (0.4)	0.3	0.49 (1.06)	0.643
3500~	0.00		0.00		0.00		0.00	
Has management responsibility								
Yes	1.08 (1.40)	0.441	−0.65 (0.85)	0.446	0.49 (0.35)	0.158	0.06 (0.91)	0.948
No	0.00		0.00		0.00		0.00	
Workplace violence								
No	−6.36 (1.26)	**< 0.001**	−5.3 (0.77)	**< 0.001**	−1.59 (0.31)	**< 0.001**	−0.53 (0.82)	0.519
Yes	0.00		0.00		0.00		0.00	

Abbreviations: *CHC* Community health center, *DP* Depersonalization, *EE* Emotion exhaustion, *PA* Personal accomplishment, *THC* Township health center

^a^GPs who have acquired associate degree or vocational diploma. An associate degree required 3 years of education in college after graduation from senior middle school (grade year 10 to year 12), or 5 years of education in college after graduation from junior middle school (grade year 7 to year 9). A vocational diploma requires 2 years of education in vocational schools after graduation from senior middle school, or 3 years of education in vocational schools after graduation from junior middle school

^b^Wages and welfares of GPs would be paid by the Chinese government public health services expenditure. GPs were unable to be freely fired off by health institutions

^c^GPs were with junior or without any technical title

^d^GPs were with middle or senior professional title

Significant values (*P* < 0.05) are presented in bold

A high professional level was positively associated with EE. Additionally, GPs who reported workplace violence had significantly higher EE scores than those without exposure to workplace violence. Being single, having a lower level of job satisfaction, and having a low professional title were associated with increased DP scores. GPs who experienced workplace violence had significantly higher DP scores than those who did not. In addition, we found that higher PA scores among GPs were associated with a higher level of job satisfaction.

Table 4 summarizes the comparisons between burnout scores and prevalence rates reported previously in some Western countries and the data from the present study.

**Table 4:** Comparison of burnout rates among our sample and previously published studies among GPs in other countries

Authors and year	Study population	Sample size	Male (%)	Age at baseline	Burnout rates	Comparable the present study data
Kirwan and Armstrong, 1995	Britain, British GPs	245	81.6	NA	Mean score of 26.1 for EE, 9.8 for DP; 36.2 for PA	EE, DP and PA: higher
Grassi and Magnani, 2000	Italy, Italian GPs	182	74.7	Mean = 42.3	High EE burnout in 32%; high DP burnout in 27%; high PA burnout in 13%	EE and DP: higher; PA: lower
Thommasenet et al., 2001	Canada, Canada GPs	131	74	Mean = 43.6	Moderate to high EE burnout in 80%; moderate to high DP burnout in 61%; moderate to high PA burnout in 44%	EE and DP: higher; PA: lower
Cathebras et al., 2004	France, French GPs	306	39.8	Mean = 46.9	5% scored high in all three dimensions	Overall burnout: higher
Goehring et al., 2005	Switzerland, Swiss primary care doctor	1755	83.6	Mean = 50.8	High EE burnout in 19%; high DP burnout in 22%; high PA burnout in 16%; 3.5% scored high in all three dimensions	EE and PA: lower; DP: higher; Overall burnout: higher
Soler et al., 2008	Europe, European GPs	1393	54.6	Mean = 45.4	High EE burnout in 43%; high DP burnout in 35%; high PA burnout in 32%; 12% scored high in all three dimensions	EE and DP: higher; PA: comparable; Overall burnout: higher
Brøndt et al., 2008	Denmark, Danish GPs	377	60.7	Mean = 51.8	2.8% scored high in allthree dimensions	Overall burnout: comparable
Lee et al., 2008	Canada, Canada Family physicians	123	62.6	Median = 47	High EE burnout in 47.9%; high DP burnout in 46.3%; high PA burnout in 17.4%	EE and DP: higher; PA: lower
Yaman and Soler, 2002	Europe, European GPs	98	59	Mean = 43.2	Mean score of 25.1 for EE, 7.3 for DP; 34.5 for PA	EE and DP: higher; PA: comparable
O’Dea et al., 2017	Ireland, Irish GPs	683	50.2	<40, 40–70	High EE burnout in 52.7%; high DP burnout in 31.6%; high PA burnout in 16.3%; 6.6% scored high in all three dimensions	EE and DP: higher; PA: lower; Overall burnout: higher
Orton et al.,2012	UK, UK GPs	564	68.5	NA	High EE burnout in 46%; high DP burnout in 42%; high PA burnout in 34%	EE and DP: higher; PA: comparable
Arigoni et al.,2009	Switzerland, Swiss GPs	141	32	NA	High EE burnout in 36%; high DP burnout in 36%; high PA burnout in 15%	EE and DP: higher; PA: lower

Note: Abbreviations: *DP* Depersonalization, *EE* Emotion exhaustion, *GP* General practitioner, *NA* Not available, *PA* Personal accomplishment

## Discussion

This is the first study to investigate the prevalence of burnout and related factors among GPs in Hubei, China. We found that 2.46% of GPs had a high level of burnout, with 24.83% scoring high for EE, 6.21% scoring high for DP, and 33.99% at a high risk of PA. Being single, having a lower level of job satisfaction, and experiencing workplace violence were significantly associated with a higher risk of burnout. Factors associated with a higher level of EE included a lower level of job satisfaction, a higher professional level, and experiences of workplace violence. Being single, having a lower level of job satisfaction, being at a lower professional level, and having experience of workplace violence were significantly associated with a higher level of DP. However, a lower level of job satisfaction was the only predictor of a higher level of PA.

### Comparison with existing literature

Compared to Western countries, our study showed lower mean EE and DP scores and a higher mean PA score. Chinese GPs were also less often categorized as having a high risk of EE and DP. Specifically, our study found that 33.99% of GPs were at high risk of a reduced sense of PA. These differences may be partly attributable to the participants’ demographic factors (age, gender, professional level, and marital status), organizational factors (healthcare system, absence of work incentives (e.g., inadequate income), work time, and career progression), the type of questionnaire, and social support. Reduced PA may reflect reduced feelings of competence and productivity at work, which may lead to a higher risk of depression [2, 31]. Thus, improving the feeling of PA among Chinese GPs is a critical challenge faced by the government of China and calls for greater efforts from health policymakers and researchers.

In line with earlier studies, our findings showed that exposure to workplace violence was associated with a significantly higher risk of burnout in the areas of EE and DP [7, 32, 33]. Workplace violence is a key occupational hazard [34] which is faced by healthcare professionals worldwide and negatively affects healthcare workers’ mental health and general well-being. It has been associated with reduced job satisfaction, commitment and efficiency, poor quality of life, increased stress, sleep disruption, and occupational burnout, and even death [35–39]. Therefore, establishing an alarm and monitoring system and improving the relationship between patients and GPs are urgently needed to reduce violence in general practice.

We observed in the present study that GPs at higher professional levels were prone to higher risk of DP and EE. In China, the higher the GP’s professional level, the heavier his workload [22]. A highly intensive workload not only affects the health of GPs, but also affects the quality of health care [40]. Patients had high expectations of higher-ranking physicians. They preferred the services of GPs with many years of experience or with higher professional titles, even for minor illnesses or self-limited conditions [41, 42]. Thus, heavy workload increases work pressure on physicians, resulting in overwork. Long-term overwork could lead to the rushed, indifferent and disrespectful management of patients, which is a major constraint in the doctor-patient relationship [41]. The above issues could result in an increased likelihood of violence among GPs. In addition, workplace violence has been identified as an important predictor of occupational burnout [43–46], which is consistent with our findings. More importantly, we found that GPs at a higher professional level were more likely to experience workplace violence (61.8% vs. 38.2%, *P* < 0.0001).

Expectedly, job satisfaction was identified as a significant predictor of burnout in this study. Previous studies have demonstrated that higher levels of burnout are associated with lower levels of job satisfaction [12, 19, 47]. This finding suggests that an improvement in job satisfaction could serve as an effective way to prevent or minimize burnout, and provides new insights for future studies on ways to improve the job satisfaction of GPs and indirectly address burnout problems.

### Limitations

This study has several limitations. First, this study used a cross-sectional study design, which precluded the evaluation of the temporality and causality of the observed relationships. Second, data were collected from participants’ self-reports; thus, recall bias was unavoidable. Third, other potential predictors of burnout and the overall health of GPs (such as work schedules, work hours, work stress, sleep quality, job control, social support, other occupational health factors, self-reported health status, and mental health) were not captured in our questionnaire. Fourth, completing the questionnaire after following a seminar on the quality of health care may have introduced bias. Finally, this study was based on the data of a cross-sectional study conducted from December 2014 to March 2015. There is a seasonal trend in the work load of GPs that may have affected the results.

### Implications for research and practice

Based on our findings, we suggest that, first, prospective studies are needed to investigate the association between the determined factors and burnout. Second, this study highlights the need for the investigation or implementation of interventions to improve GPs’ well-being or strategies to reduce work pressure on GPs in China’s primary healthcare settings. Third, a previous study [48] showed that new medical graduates are susceptible to burnout. Additional studies investigating the work demands, job satisfaction, burnout, and turnover intentions of new GPs could provide a valuable insight. Finally, investigating the potential impact of burnout on GPs’ work performance, the quality of patient care delivery, and family life would provide important insights.

## Conclusions

In conclusion, burnout among GPs, especially single GPs, GPs with lower job satisfaction, and those with exposure to workplace violence, is prevalent in Hubei, China. In total, 25% of GPs suffered from high levels of EE, 6% suffered from high DP and 34% suffered from reduced PA.

## Data Availability

Data may be made available by contacting the corresponding author.

## Abbreviations

CHC: Community health center
CHS: Community health service
DP: Depersonalization
EE: Emotional exhaustion
GP: General practitioner
PA: Personal achievement
PHI: Primary healthcare institution
THC: Township health center
