# Correction: Prevalence of burnout and associated factors among general practitioners in Hubei, China: a cross-sectional study

**DOI:** 10.1186/s12889-022-14050-7

**Published:** 2022-10-12

**Authors:** Yong Gan, Heng Jiang, Liqing Li, Yudi Yang, Chao Wang, Jianxin Liu, Tingting Yang, Sampson Opoku, Sai Hu, Hongbin Xu, Chulani Herath, Yuanyuan Chang, Pengqian Fang, Zuxun Lu

**Affiliations:** 1 Department of Social Medicine and Health Management, School of Public Health, Tongji Medical College, Huazhong University of Science and Technology, No. 13 Hangkong Road, Wuhan, 430030 Hubei China; 2 Centre for Alcohol Policy Research, School of Psychology and Public Health, La Trobe University, Melbourne, Victoria Australia; 3 Centre for Health Equity, Melbourne School of Population and Global Health, University of Melbourne, Melbourne, Victoria Australia; 4 Department of Management Science and Engineering, School of Economics and Management, Jiangxi Science and Technology Normal University, Nanchang, Jiangxi China; 5 Department of Nutrition, People’s Hospital of Henan Province, Zhengzhou, Henan China; 6 Department of Medical Records and Statistics, Union Hospital, Tongji Medical College, Huazhong University of Science and Technology, Wuhan, Hubei China; 7 Department of Psychology and Counselling, Faculty of Health Sciences, The Open University of Sri Lanka, Nawala, Nugegoda, Sri Lanka; 8 Academy of Health Policy and Management, School of Medicine and Health Management, Tongji Medical College, Huazhong University of Science and Technology, No. 13 Hangkong Road, Wuhan, Wuhan, 430030 Hubei China; 9 Nanjing Medical University, Nanjing, Jiangsu China

**Correction: BMC Public Health 19, 1607 (2019)**

**https://doi.org/10.1186/s12889-019-7755-4**

In the original publication [1] the authors omitted to state the contribution of author Chulani Herath. The Author Contributions section should read:

- YG and ZXL conceived and designed the study. YDY, CW, JXL, TTY, SH, HBX, and YYC participated in the acquisition of data. YG analyzed the data. HJ, LQL, and PQF gave advice on methodology. YG wrote the draft of the paper. CH contributed to revising the paper. All authors contributed to writing, reviewing or revising the paper and read and approved the final manuscript. ZXL is the guarantors of this work and has full access to all the data in the study and takes responsibility for its integrity and the accuracy of the data analysis. All authors read and approved the final manuscript.

## References

[1] Gan Y (2019). Prevalence of burnout and associated factors among general practitioners in Hubei, China: a cross-sectional study. BMC Public Health.

